# Imaging manifestations of hereditary hemorrhagic telangiectasia with pulmonary arterial hypertension: a case report

**DOI:** 10.3389/fcvm.2025.1548130

**Published:** 2025-03-21

**Authors:** Guangmei Qin, Siqi Chen, Fuling Huang, Liupei Mo, Kai Li

**Affiliations:** Department of Radiology, The First Affiliated Hospital of Guangxi Medical University, Nanning, Guangxi, China

**Keywords:** hereditary hemorrhagic telangiectasia, imaging, 4D flow, pulmonary arterial hypertension, case report

## Abstract

Hereditary hemorrhagic telangiectasia (HHT) is a rare autosomal dominant disorder. Pulmonary arterial hypertension (PAH) is an uncommon complication (affecting <1% of HHT patients). Here, we report the clinical and imaging findings of a rare case of HHT complicated by PAH in a 41-year-old woman. The patient experienced recurrent exertional dyspnea for over 1 year, accompanied by chest tightness and pain, coughing, and production of white mucus. Her medical history included recurrent epistaxis and bilateral lower extremity edema. Due to persistent symptoms, she was hospitalized for further evaluation. Imaging revealed multiple dilated, tortuous vessels and arteriovenous fistulas in both lower lung lobes and the liver. Additionally, myocardial edema and fibrosis were observed in the ventricular insertion points, interventricular septum, right ventricular inferior wall and left ventricular free wall. Reduced pulmonary artery peak flow velocity, maximal flow, and mean wall shear stress (mWSS) were noted. Right heart catheterization confirmed pre-capillary PAH, and genetic testing identified an *ACVRL1* mutation. Symptomatic supportive care was provided during hospitalization. We discussed the relationship between PAH and HHT as well as the characteristics of both conditions.

## Case presentation

A 41-year-old female presented with over a year of recurrent exertional dyspnea, worsening with activities like climbing stairs but improving with rest. She also reported chest tightness, pain, cough, and white mucoid sputum. She had a history of recurrent epistaxis and bilateral lower limb edema. Her family history included similar symptoms in her mother and recurrent epistaxis in her father and sister, with the latter also having PAH. Physical examination revealed oral telangiectasias, coarse lung sounds, an accentuated split P2, a 3/6 diastolic murmur at the L3–4 area, and moderate pitting edema in both lower limbs.

The patient presents with a complex clinical scenario involving both cardiovascular, pulmonary, and hepatic manifestations, likely stemming from PAH and associated right heart failure. Her laboratory investigations were remarkable as follow:

According to the laboratory findings, there was compelling evidence of both hepatic and renal dysfunction. The elevated levels of N-terminal pro brain natriuretic peptide (NT-proBNP) pointed towards cardiac insufficiency. But the coagulation profile, rheumatoid factor, as well as qualitative and quantitative assays for autoimmune antibodies, alongside quantitative HIV antibody testing, revealed no anomalies. Laboratory data in detail are shown in Supplementary Table 1.

The electrocardiogram showed ST-T changes and right axis deviation (Supplementary Figure 1). Pulmonary function tests indicated moderate obstructive ventilatory dysfunction, mild diffusion impairment, and an increased residual volume/total lung capacity ratio. Echocardiography revealed significant enlargement of the right atrium and ventricle, severe tricuspid regurgitation, elevated pulmonary artery pressure (approx. 51 mmHg), and a small pericardial effusion. No shunt flow was demonstrated between the atrial or ventricular chambers (Supplementary Figure 2).

Right heart catheterization confirmed precapillary PAH with:

- Pulmonary artery pressure (PAP): 73/50 (57) mmHg
- pulmonary artery wedge pressure (PAWP): 17/7 (11) mmHg
- Pulmonary vascular resistance (PVR): 7.9 Woods units
- Cardiac index (CI): 3.7 L/min/m^2^

Doppler ultrasound revealed hepatic congestion likely due to heart failure, along with a dilated portal vein. Color Doppler flow imaging shows hepatic veins display alternating red. Contrast-enhanced liver ultrasound suggests the hepatic arteriovenous fistula and inferior vena cava hypertension.

**Figure 1:**
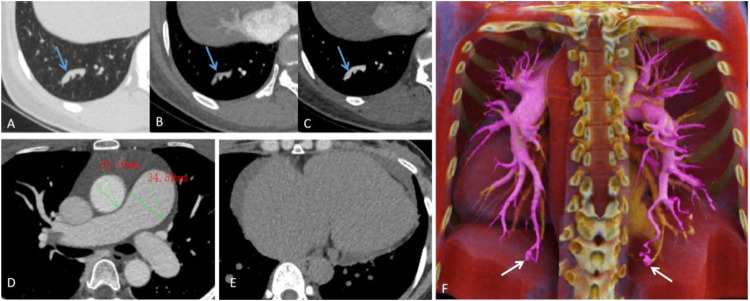
**(A–C)** Lung window, pulmonary arterial phase, and pulmonary venous phase show tortuous and dilated vascular shadows (the blue arrows), with a connection between the pulmonary artery and pulmonary vein. **(D)** Pulmonary vein phase images reveal dilation of the main pulmonary artery (approx. 34.59 mm in diameter) and its branches, compared to an aortic diameter of about 26.10 mm at the same level. **(E)** Mediastinal window images show cardiac enlargement, mainly involving the right atrium and ventricle, with a ring-shaped pericardial effusion present. **(F)** Maximum intensity projection image shows thickened, tortuous feeding arteries (The purple-colored vessels), an aneurysmal sac (The white arrows), and draining veins (The yellow-colored vessels).

CT pulmonary angiography (CTPA) revealed pulmonary arteriovenous fistulas in the posterior basal segments of both lower lobes, with enlarged feeding arteries, aneurysmal sacs, and draining veins. Signs of pulmonary arterial hypertension and a small pericardial effusion were also noted (Figure 1).

**Figure 2:**
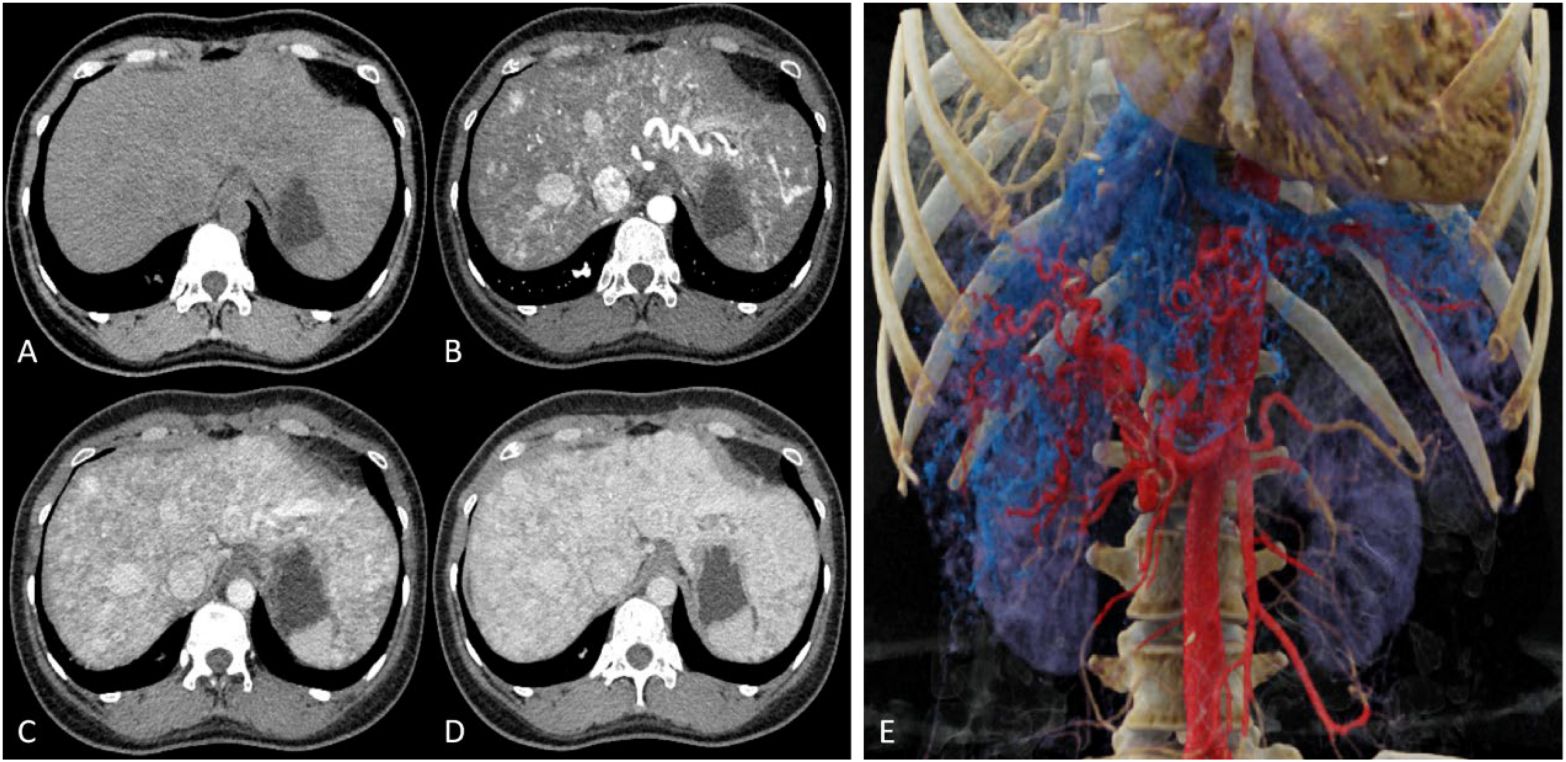
**(A)** Plain CT reveals multiple tortuous, enlarged low-density vascular shadows in the liver. **(B)** In the arterial phase, multiple tortuous and thickened arteries and veins can be seen within the liver. **(C)** The portal venous phase and **(D)** equilibrium phase indicate uniform liver enhancement with mildly dilated intrahepatic bile ducts. **(E)** 3D reconstruction images from CTV display tortuous, enlarged arteries and veins, along with dilated hepatic veins and superior mesenteric veins.

Upper abdominal CT (plain, contrast, CTV) revealed a hepatic arteriovenous fistula, dilated hepatic veins and superior mesenteric vein, and mild intrahepatic bile duct dilation (Figure 2). There were no observed signs of portal hypertension, such as esophageal and gastric varices, ascites, splenomegaly, or other related manifestations. Lower extremity CTA and CTV showed no significant abnormalities.

**Figure 3:**
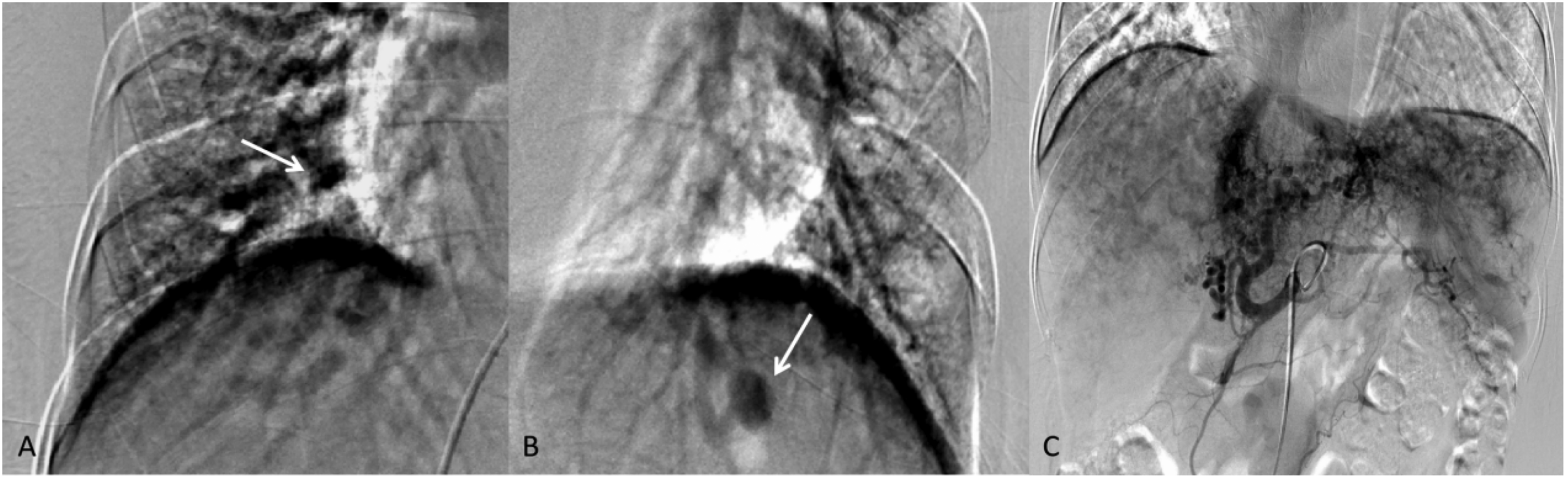
**(A,B)** Mild arteriovenous fistula signs in both lower lobes (the white arrows). **(C)** The left lobe hepatic artery shows abnormal disarray with early visualization of the left hepatic vein and inferior vena cava during the mid-arterial phase, indicating a left hepatic artery-vein fistula.

Pulmonary angiography indicates mild arteriovenous fistulas in the pulmonary arteries of both lower lobes. Hepatic artery angiography revealed a left hepatic artery-vein fistula. Superior mesenteric artery angiography appeared normal (Figure 3).

**Figure 4:**
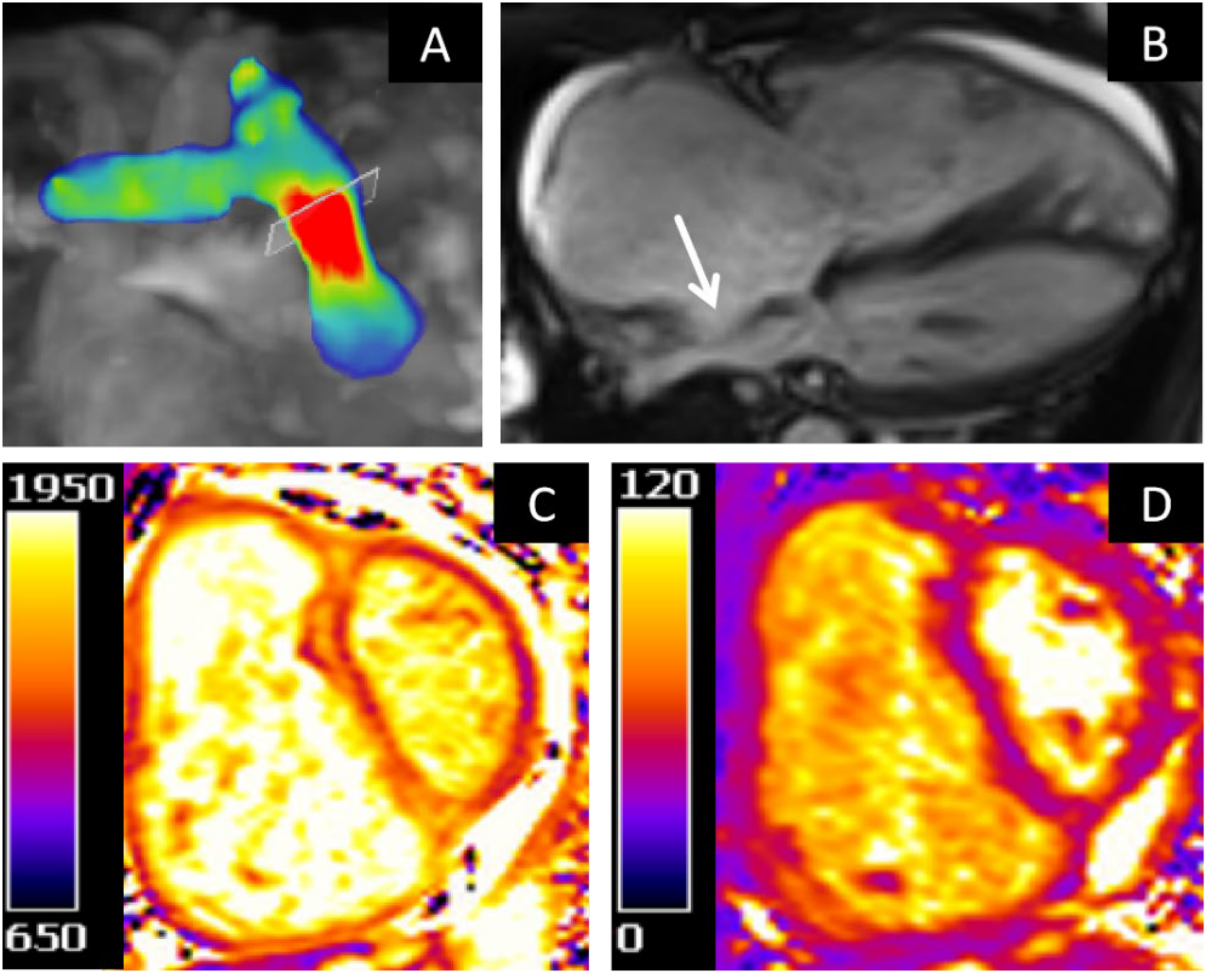
**(A)** 4D flow velocity mapping. **(B)** Cine four-chamber view. **(C)** T1 mapping of the basal segment. **(D)** T2 mapping of the basal segment.

Cardiac MRI demonstrates enlargement of the right atrium and ventricle, with bulging of the interatrial septum toward the left atrium. Functional assessment revealed that the left ventricular ejection fraction (65.12%), left ventricular end-diastolic volume (95.01 ml), and left ventricular end-systolic volume (33.14 ml) were all within normal ranges. In contrast, the right ventricular end-diastolic volume (267.46 ml) and end-systolic volume (169.53 ml) were significantly elevated, accompanied by a markedly reduced right ventricular ejection fraction of 26.63%. 4D flow imaging showed reduced peak flow velocity (1.1 m/s), maximum flow (14.64 L/min), and mWSS (0.141 Pa) in the pulmonary artery. Elevated T1 (1,271–1,604 ms) and T2 (41–53 ms) mapping values were noted in the ventricular insertion points, right ventricular inferior wall, interventricular septum, and left ventricular free wall (Figure 4).

Genetic screening identified a heterozygous ACVRL1 gene mutation, supporting the Genetic related PAH diagnosis. Among her family members, only her sister and her two children underwent genetic testing, and no positive gene mutations were found.

Following a diagnosis of pulmonary arterial hypertension (PAH), the patient was initiated on combination therapy with a dual endothelin receptor antagonist and a type 5 phosphodiesterase inhibitor, resulting in significant symptomatic improvement. During follow-up surveillance, the patient experienced an episode of substernal chest pain accompanied by diaphoresis, with recurrent episodes persisting for 10–30 min per occurrence and symptom recurrence within a 1-h timeframe. Laboratory tests showed NT-proBNP of 588.400 pg/ml (normal 0–300 pg/ml) and D-dimer of 0.649 mg/L (normal 0–0.3 mg/L). while serum troponin I and creatine kinase-MB isoenzyme levels remained within normal ranges. No specific therapeutic interventions were administered. Currently, the patient remains in stable condition with occasional episodes of cough and epistaxis (Supplementary Figure 3).

## Discussion

Hereditary hemorrhagic telangiectasia (HHT) is an autosomal dominant vascular disease characterized by multiple vascular malformations (VMs). Its pathogenic genes mainly include ENG, ACVRL1, GDF2, and SMAD4, which all affect the transforming growth factor-β (TGF-β) signaling pathway, leading to abnormal vascular development. 70% of hereditary pulmonary arterial hypertension (HPAH) is caused by mutations in the bone morphogenetic protein receptor type II (BMPR2) gene, and less than 1% of HHT patients develop HPAH caused by mutations in the ACVRL1 gene (1). HHT related HPAH is mostly post capillary pulmonary arterial hypertension, with only a small percentage of HHT patients experiencing pre capillary pulmonary arterial hypertension (2). The ACVRL1 gene mutation has been confirmed to be closely associated with the comorbidity of HHT with pulmonary arterial hypertension (PAH) (3), and this gene mutation is more likely to induce hepatic arteriovenous malformations (HAVMs) (1). Both PAH and HAVMs are associated with poorer prognosis in HHT patients (4–6). We report the clinical and imaging characteristics of a patient with HHT complicated by PAH (HHT-PAH), which is associated with an ACVRL1 mutation, and integrate literature case data from 36 cases with ACVRL1 mutation (Supplementary Table 2), hoping to improve people's understanding of this disease.

The age range of the literature research cohort is from 14 months to 70 years old (mean 33.3 years old), with females accounting for 76% (25/33) and males accounting for 24% (8/33). During the follow-up period, there were a total of 10 deaths, including 4 deaths from heart failure, 1 death from hemorrhagic shock, 1 death from pulmonary arteriovenous malformation rupture, 1 death during abdominal perioperative period, and the remaining 3 deaths with unclear causes of death. The imaging features of HHT-PAH integrate the dual pathological manifestations of HHT and PAH. The core imaging feature of HHT is characterized by abnormal development of multiple vascular systems. (1) Pulmonary arteriovenous malformations (PAVMs): characterized by multiple occurrences in the bilateral basal regions, early pulmonary capillary dilation may present as solid or ground glass nodules, which need to be differentiated from simple pulmonary nodules (1, 7, 8). (2) Liver involvement: CT/MRI can display diffuse arteriovenous shunting, hepatic artery dilation with increased blood flow, and in some cases, focal nodular hyperplasia and portal hypertension like “pseudo cirrhosis” signs may occur (9). (3) Central nervous system: relatively rare, cerebral arteriovenous malformations or bilateral basal ganglia T1WI hyperintensities have been reported in HHT patients. Distinguish from metabolic encephalopathy (10–12). (4) A few researchers have reported pancreatic arteriovenous malformations in HHT patients (13, 14). Disregarding clinical features to diagnose HHT is a lack of consideration, which may lead to misdiagnosis or missed diagnosis. Therefore, when HHT related symptoms occur, we need to comprehensively consider the patient's condition and make an accurate diagnosis according to the guidelines. The imaging features of PAH include: (1) Pulmonary vascular remodeling: main pulmonary artery dilation (diameter ≥29 mm) and main pulmonary artery/ascending aorta diameter ratio >1.0. (2) Compensation and decompensation of right ventricular function: right ventricular hypertrophy, dilation (increased right ventricular end diastolic volume), flattening or deviation of interventricular septum, decreased right ventricular ejection fraction (15). (3) Myocardial tissue characteristics: There may be myocardial edema, fibrosis, etc. in the right ventricular septal insertion area, interventricular septum, right ventricular free wall, or left ventricular free wall, manifested as delayed contrast enhancement (DCE) positive or increased original T1/T2 or ECV values. These abnormal changes may be related to poor outcomes of PAH (16–18). (4) 4D Flow hemodynamic markers: formation of main pulmonary artery eddies, early retrograde flow during contraction, pulmonary artery velocity, maximum flow rate, and decreased wall shear stress (WSS) (19–22). This patient presents typical HHT-PAH imaging features (pulmonary/hepatic arteriovenous malformation, main pulmonary artery dilation, and right ventricular remodeling), but due to economic factors, DCE examination was not performed to evaluate myocardial tissue characteristics.

The right heart catheterization (RHC) confirmed the diagnosis of precapillary PAH in the patient. The etiology can be traced back as follows: (1) The patient had no history of long-term use of PAH-associated medications (e.g., chemotherapeutic agents) or exposure to toxins (e.g., cocaine), thereby ruling out drug- and toxin-induced PAH. (2) Serological testing revealed negative results for autoimmune antibodies and rheumatoid factor, and there was no evidence of human immunodeficiency virus infection or schistosomiasis exposure. Upper abdominal computed tomography did not indicate portal hypertension, and echocardiography and cardiac magnetic resonance imaging showed no cardiac defects or left ventricular disease (with normal left ventricular ejection fraction and no valvular abnormalities). These findings collectively excluded disease-associated PAH and left heart disease-induced pulmonary hypertension. (3) Coagulation profiles were within normal limits, and CT pulmonary angiography did not reveal any signs of chronic thromboembolism or pulmonary venous occlusive disease, thus excluding these conditions as potential causes. Additionally, no primary lung diseases such as interstitial lung disease or chronic obstructive pulmonary disease were identified, thereby further ruling out pulmonary arterial hypertension caused by lung diseases or hypoxia. Further analysis of the clinical and radiographic features: recurrent epistaxis, mucocutaneous telangiectasia, radiologic evidence of PAVMs and HAVMs, along with the detection of an ACVRL1 gene mutation, align with the Curaçao diagnostic criteria (23). This constellation of findings strongly supports the diagnosis of HHT-PAH. During the follow-up process, the patient experienced chest pain, possibly due to increased oxygen consumption in the enlarged right ventricle or myocardial ischemia caused by right ventricular dilation compressing the left coronary artery (24).

The pathogenesis of HHT-PAH exhibits significant heterogeneity and can be divided into two main types: (1) High cardiac output (CO) PAH: Systemic hyperdynamic circulatory status [CO >8 L/min or cardiac index (CI) ≥4 L/min/m^2^] caused by HAVMs is an important mechanism grouping criterion for HHT-PAH (25), especially in patients with ACVRL1 mutations. This type of patient's RHC shows a significant increase in CO, elevated pulmonary capillary wedge pressure (PAWP), and normal pulmonary vascular resistance (PVR). (2) Pre capillary PAH: The characteristics of RHC are elevated mean pulmonary artery pressure (mPAP), low CO, normal PAWP, and high PVR (26). According to the results of the right heart catheterization, the patient was diagnosed with pre capillary PAH. It is worth noting that when high cardiac output PAH occurs, the true PVR may be underestimated. Liu et al. reported a female patient with HHT accompanied by pre capillary PAH. She developed high CO and high PAWP after receiving treatment (27). According to [PVR = (mPAP−PAWP)/CO] (25), we speculate that the potential mechanism may be that shunting significantly increases CO, and even if mPAP is mildly elevated, PVR may be underestimated, masking the true pulmonary vascular lesions. For HHT-PAH secondary to elevated cardiac output, management focuses on addressing high-output states, whereas in HHT-associated precapillary PAH, targeted vasodilatory therapies including endothelin-1 receptor antagonists and phosphodiesterase-5 inhibitors constitute the recommended therapeutic approach (28). Endothelin receptor antagonists promote vasodilation by blocking endothelin-1, while phosphodiesterase-5 inhibitors achieve the same effect by inhibiting the degradation of cyclic guanosine monophosphate (29). However, vasodilators are associated with an increased risk of elevated shunt flow in patients with HHT. Currently, only a few case reports describe the response to pulmonary vasodilators in PAH patients with HHT and ACVRL1 gene mutations. Yokokawa et al. reported a case involving an HHT-PAH patient with an ACVRL1 mutation who experienced a reduction in mean pulmonary artery pressure following treatment with vasodilators (30). Our patient's symptoms improved after receiving treatment with a combination of endothelin receptor antagonists and phosphodiesterase inhibitors. It should be noted that for pulmonary arterial hypertension patients with concomitant hyperdynamic circulation, PAH specific treatment may induce right heart failure by further reducing systemic vascular resistance (25). Therefore, precise classification through RHC is necessary before treatment.

The etiology of pulmonary arterial hypertension is complex. Standardized etiological screening and early identification of risk factors are key to improving prognosis. For patients with HHT-PAH, it is important to be vigilant about its dual mechanisms to avoid misdiagnosis as idiopathic PAH. Select the correct treatment plan based on different hemodynamic characteristics.

## Future directions

Establishing an international HHT-PAH registry to correlate imaging findings with outcomes across genotypes. Developing AI-integrated advanced imaging for quantifying visceral arteriovenous malformation (AVM) shunts and PAH severity. Prospectively evaluating targeted drug-embolization synergies to establish stratified management guidelines.

## Conclusion

We describe a rare case of hereditary hemorrhagic telangiectasia (HHT) with an ACVRL1 mutation complicated by pulmonary arterial hypertension (PAH), delineating its diagnostic trajectory and multimodal imaging features, including CT pulmonary angiography, echocardiography, and cardiac magnetic resonance imaging. This case provides novel insights into the pathophysiological continuum between pulmonary vascular remodeling and HHT, particularly underscoring the paucity of imaging-documented genotype-phenotype correlations in the existing HHT literature.

## Data Availability

The datasets presented in this study can be found in online repositories. The names of the repository/repositories and accession number(s) can be found in the article/Supplementary Material.
