# Supplementary material for: Imaging manifestations of hereditary hemorrhagic telangiectasia with pulmonary arterial hypertension: a case report
Source: Front Cardiovasc Med. 2025 Mar 21;12:1548130. doi: 10.3389/fcvm.2025.1548130 (PMC11968766; doi:10.3389/fcvm.2025.1548130)
Supplement: Supplementary file 1 [file Table1.pdf]

Supplementary Table 1. The Laboratory Findings on Admission.

Parameters	Value	Reference interval	Unit	Parameters	Value	Reference interval	Unit
Blood count				Coagulation function	normal		
Red blood cell	4.49	3.8-5.1	$\times 10^{12}$/L	Antibody Testing	normal		
Hemoglobin	125	115-150	g/L	HBsAg	0	0-0.05	IU/ml
Liver function				HBsAb	439.28	0-10	mIU/ml
Total bilirubin	43	0-21	μ mol/L	HBeAg	0	0-0.05	PElu/ml
Direct bilirubin	27.2	0-6.8	μ mol/L	HBeAb	0.34	0-0.2	PElu/ml
IBiL	15.8	3.1-14.3	μ mol/L	HBcAb	2.94	0-0.9	PElu/ml
Total protein	63.8	65-85	g/L	HCV Ab	0.1	0-1	S/CO
Albumin	33.8	40-55	g/L	HIV Ab	0.16	0-1	S/CO
GGT	140	7-45	U/L	BLD	+3	Negative	Cell/uL
TBA	103.4	0-10	μ mol/L	Urobilinogen	+1	Negative	UBG
AST	34	13-35	U/L	ApoA-I	0.87	1.2-1.6	g/L
ALT	16	7-40	U/L	ApoB	0.46	0.8-1.05	g/L
ALP	162	35-100	U/L	Potassium	3.46	3.5-5.3	mmol/L
Prealbumin	105.9	180-350	mg/L	Magnesium	0.7	0.75-1.02	mmol/L
Cholinesterase	3799	5000-12000	U/L	Creatine kinase	72	40-200	U/L
Renal function				CK-MB	16	0-25	U/L
Creatinine	98	41-73	μ mol/L	LDH	280	120-250	U/L
Uric acid	581	155-357	μ mol/L	RF	2.5	≤18	IU/ml
Bicarbonate	20.3	22-29	mmol/L	cTnI	0.007	0.000-0.029	ng/ml
Cystatin C	1.15	0.59-1.03	mg/L	Thyroid Function	normal		
NT-proBNP	748	0-125	pg/ml				

Notes:IBiL: Indirect bilirubin, GGT:Gamma-Glutamyl Transferase, TBA:Total Bile Acids, AST: Aspartate Aminotransferase, ALT: Alanine Aminotransferase, ALP: Alkaline Phosphatase, NT-proBNP: N-terminal pro brain natriuretic peptide, The coagulation function includes Prothrombin Time, Activated Partial Thromboplastin Time, International Normalized Ratio, Fibrinogen, and D-dimer.Antibody testing includes Antinuclear Antibody, Anti-double-stranded DNA Antibody, Anti-U1 RNP Antibody, Anti-Smith Antibody, Anti-Sjögren's Syndrome A Antibody, Anti-Ro-52 Antibody, Anti-Sjögren's Syndrome B Antibody, Anti-Topoisomerase I Antibody, Anti-Jo-1 Antibody, Anti-Centromere Protein B Antibody, Anti-double-stranded DNA Antibody, Anti-Nucleosome Antibody, Anti-Histone Antibody, Anti-Ribosomal P Protein Antibody, Perinuclear Anti-Neutrophil Cytoplasmic Antibody, Cytoplasmic Anti-Neutrophil Cytoplasmic Antibody, Atypical Anti-Neutrophil Cytoplasmic Antibody, Anti-Myeloperoxidase IgG Antibody, Anti-Proteinase 3 IgG Antibody, Anti-Cardiolipin IgG Antibody, Anti-Cardiolipin IgM Antibody, Anti-Cardiolipin IgA Antibody, and Anti-Cyclic Citrullinated Peptide Antibody. HBsAg: Hepatitis B Surface Antigen, HBsAb: Hepatitis B Surface Antibody, HBeAg: Hepatitis B e Antigen, HBeAb: Hepatitis B e Antibody, HBcAb: Hepatitis B Core Antibody, HCV Ab: Hepatitis C Virus Antibody, HIV Ab: Human Immunodeficiency Virus Antibody, BLD: Occult Blood in Urine, ApoA-I: Apolipoprotein A-I, ApoB: Apolipoprotein B, CK-MB: Creatine Kinase-Myocardial Band, LDH:

Lactate Dehydrogenase, RF: Rheumatoid Factor, cTnI: Serum Cardiac Troponin I, Thyroid function includes free T3, free T4, and thyroid stimulating hormone.
