# Supplementary material for: Imaging manifestations of hereditary hemorrhagic telangiectasia with pulmonary arterial hypertension: a case report
Source: Front Cardiovasc Med. 2025 Mar 21;12:1548130. doi: 10.3389/fcvm.2025.1548130 (PMC11968766; doi:10.3389/fcvm.2025.1548130)
Supplement: Supplementary file 2 [file Table2.pdf]

Supplementary Table 2. Characteristics and prognosis of hereditary hemorrhagic telangiectasia and pulmonary hypertension patients with ACVRL1 mutation in the literature

References	Caseload	Age at HHT Diagnosis (years)	Sex	Life cycle	Cause of death	Mutation type	Image findings
(1)	1	30	fm	28 years	-	c.1451G>T (p.Arg484Leu)	PAD; HAVMs; RHE
(2)	1	70	m	-	-	-	PAD; HAVMs; HVD; DIVC; RHE; IF; Bilbilateral basal segment T1 hyperincreased
(3)	1	45	fm	6 months	heart failure	c. 1232G>A, p. Arg411Gln	PAD; HVD; RHE; TR; Polyplasma membrane cavity effusion
(4)	1	28	fm	-	-	NM_000020.3: c.698C > G (p.S233W) at chr12:52308295	PAVMs; RHE
(5)	1	5	fm	1 year	heart failure	a c.1121G>A	HVD; HAVMs
(6)	1	58	m	-	-	c.355+ C>T (rs2071218)	Right lower lobe PAVMs
(7)	1	17	fm	21 years	heart failure	-	PAD; RHE
(8)	1	47	fm	-	-	982 CNT, His 328 Thr	HAVMs; RHE; TR
(9)	1	48	fm	-	hemorrhagic shock	-	HVD; HAVMs; Hepatomegaly;RHE
(10)	1	10	m	-	-	c.470T>C (p.Leu157Pro)	PAD;RHE; IF; TR
(11)	1	51	fm	-	-	c.925G＞A	left upper lobe PAVMs; HAVMs; RHE; TR
(12)	1	37	fm	-	-	c.926G＞T; p.Gly309Val	PAVMs; HAVMs; Pancreatic arteriovenous malformation; RHE; IF
(13)	3	9/30/42	fm	1-/ 37 ears/ 3-	-/abdominal operation/-	c.595G>C (p.A199P)	PAD; HAVMs; Pancreatic arteriovenous malformation; RHE; TR/ HAVMs; RHE; TR/ HAVMs; Pancreatic arteriovenous malformation; RHE

(14)	1	57	fm	-	-	-	PAVMs; HAVMs; FNH; Hepatocirrhosis; RHE; TR
(15)	1	62	fm	-	-	c.1358del (p.N453T)	HAVMs; RHE; TR
(16)	1	38	fm	69 months	NK	c.1388del,p.Gly463AlafsX2 within exon 10	HAVMs; FNH; Aneurysm of splenic artery
(17)	3	4/16/17	NK	-	-	ACVRL1 Missense mutation* Exon 7 L273P (c. T818C), ENG Substitution: intron 12 1742-72T.C/ ACVRL1 Missense mutation* Exon 8 A352D (c. C1055A).	PAD; PAVMs
(18)	1	17	fm	-	-	c.1196G > T, p.Trp399Leu	RHE; TR
(19)	1	63	fm	-	-	-	HAVMs; Intra-hepatic cholangioma; Diffuse bile duct necrosis
(20)	1	29	fm	18 months	PAVMs break	exon 9, c.1280A.T, p.Asp427Val	Right lower lobe PAVMs; HAVMs
(21)	1	63	m	-	-	c.1121G>A (p.Arg374Gln)	Not described
(23)	1	43	fm	-	-	c.1451G/A	FNH; RHE
(24)	1	4	fm	-	-	-	Multiple pulmonary nodules
(25)	1	34	fm	-	-	-	PAVMs; Pleural effussion
(26)	1	1.2	m	-	-	c.1450C>T; p.Arg484Trp	PAD; RHE
(27)	3	38，40，38	fm	180 months/ 247 months/ 45 months	heart failure	-	PAVMs; HAVMs; HVD; RHE; Portal hypertension
(28)	4	8/20/43/18	3fm1m	9/8/2years	NK	1450CwT, 1450_1451insG；1435CwT；1120CwT；	PAVMs; HAVMs

						1385CwG	

pour：fm: female; m: man; NK: Not Know; PAD: Pulmonary artery dilation；RHE: Right heart enlargement；HAVMs: Hepatic arteriovenous malformations; IF: Interval flattening; HVD: Hepatic vascular dilation; DIVC: Dilation of inferior vena cava; TR: Triapphal regurgitation; PAVMs: Pulmonary arteriovenous malformations
