# Supplementary material for: Imaging manifestations of hereditary hemorrhagic telangiectasia with pulmonary arterial hypertension: a case report
Source: Front Cardiovasc Med. 2025 Mar 21;12:1548130. doi: 10.3389/fcvm.2025.1548130 (PMC11968766; doi:10.3389/fcvm.2025.1548130)
Supplement: Supplementary file 3 [file Image1.pdf]

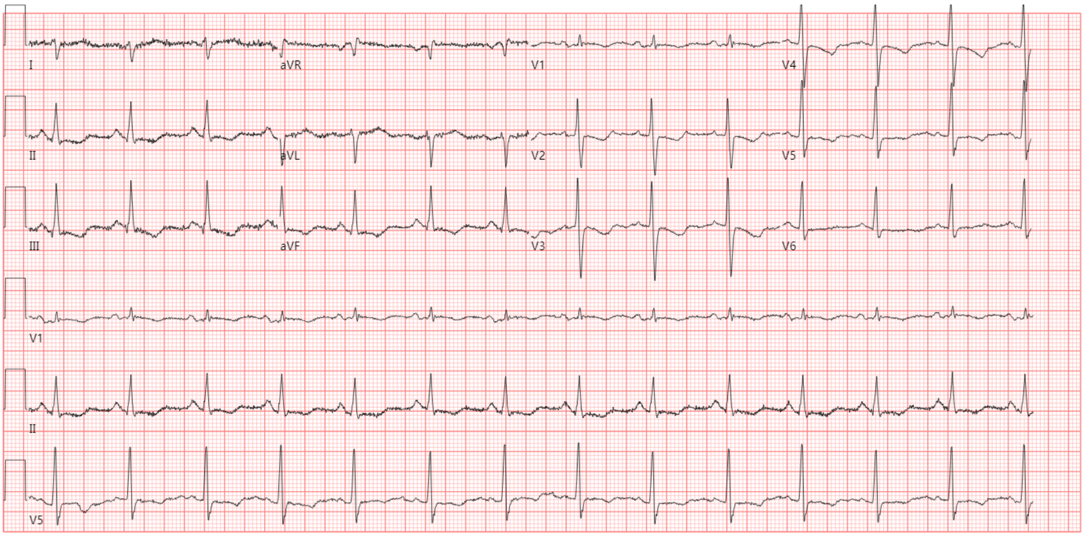

Supplementary Figure 1. Electrocardiogram. The electrocardiogram showed sinus rhythm at 84 beats per minute with ST-T changes and right axis deviation.
