# Supplementary material for: Imaging manifestations of hereditary hemorrhagic telangiectasia with pulmonary arterial hypertension: a case report
Source: Front Cardiovasc Med. 2025 Mar 21;12:1548130. doi: 10.3389/fcvm.2025.1548130 (PMC11968766; doi:10.3389/fcvm.2025.1548130)
Supplement: Supplementary file 4 [file Image2.pdf]

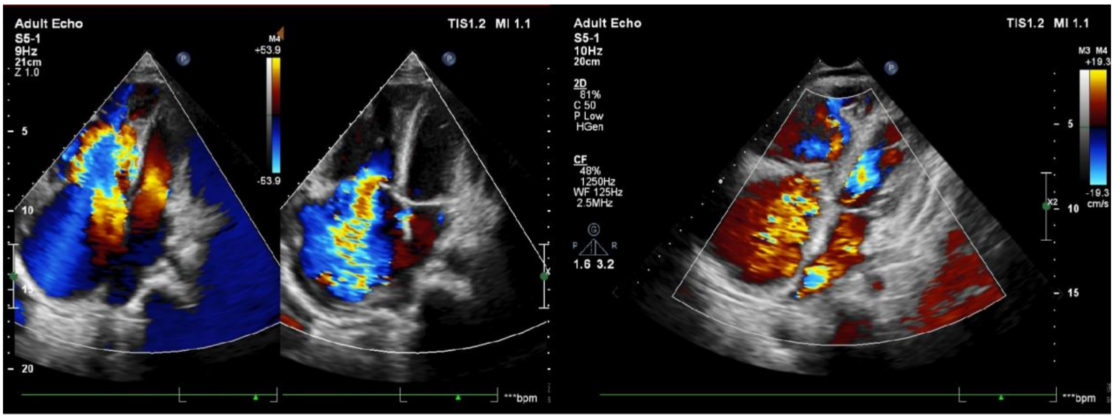

Supplementary Figure 2. Echocardiography：Echocardiographic evaluation demonstrated marked enlargement of the right atrium and ventricle while maintaining normal left atrial and ventricular dimensions, accompanied by Doppler-documented severe tricuspid valve regurgitation. The continuity of the interventricular septum and interatrial septum was intact with no detectable shunt signals observed, effectively ruling out shunt-type congenital heart disease.
