# Supplementary material for: Imaging manifestations of hereditary hemorrhagic telangiectasia with pulmonary arterial hypertension: a case report
Source: Front Cardiovasc Med. 2025 Mar 21;12:1548130. doi: 10.3389/fcvm.2025.1548130 (PMC11968766; doi:10.3389/fcvm.2025.1548130)
Supplement: Supplementary file 5 [file Image3.pdf]

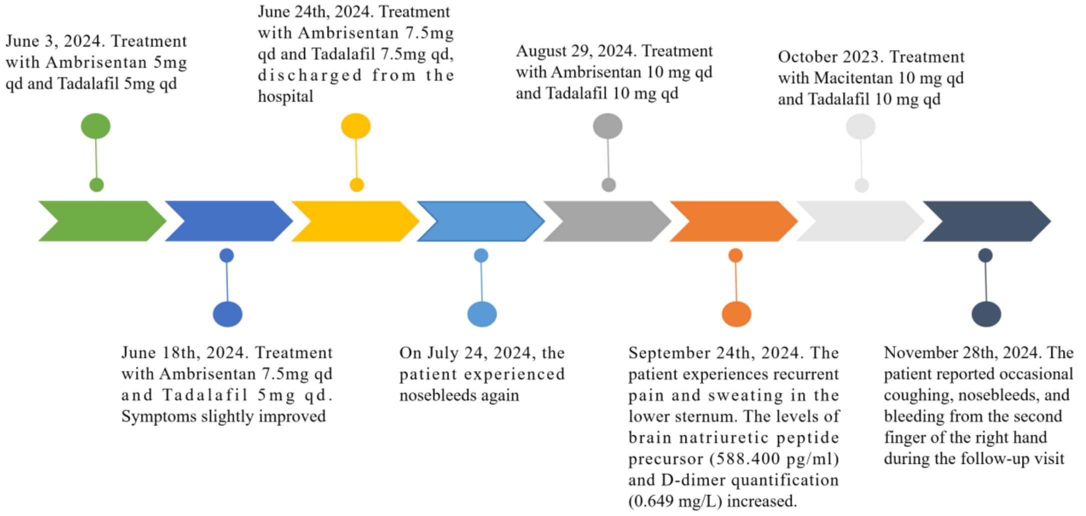

Supplementary Figure 3. Case report timeline description. The active compound name of Ambrisentan is (+)-(2S)-2-[(4,6-dimethylpyrimidin-2-yl)oxy]-3-methoxy-3,3-diphenylpropanoic acid. qd: Once a day.
